# A Fair Chance for the Panel System

**Published:** 1923-03

**Authors:** R. W. Harris


### A FAIR CHANCE FOR THE PANEL SYSTEM.

**By R. W. HARRIS.**

On the outcome of the Guildhall Conference Dr. Brackenbury and his colleagues of the Insurance Acts Committee and the representatives of the Approved Societies, the Insurance Committees, and the chemists are warmly to be congratulated. Equally a subject for congratulation is the spirit in which—disregarding the silly suggestions that the doctors had some sinister motive in calling this Conference—these several bodies came together and carried out the proceedings.

### A Better Understanding.

If the Conference were to succeed this could clearly not be because of the emergence, from such a large body, of concrete proposals on points of detail for the betterment of the medical service for the insured. This was out of the question. What might be hoped for was, firstly, the creation of a better understanding between bodies which, notwithstanding their identity of purpose, had somehow got into the habit of long-range firing at one another, and, secondly, the setting up of a small permanent committee which would meet from time to time and formulate definite proposals for the improvement of the service. Both these objects were attained. The small Committee which has been set up will get away from the vague generalities of the platform and the Press and get down to the tin tacks. It is to be hoped that it will from time to time invite the assistance of those persons who are so ready to decry this or that feature of the service—for instance, the Medical Service Sub-Committee procedure—and seem, perhaps wrongly, to be so singularly barren of workable suggestion for amendment. It is further to be hoped that they will find some way of dealing faithfully with such monstrous mis-statements as that which appeared in a daily paper, shortly after the Conference, that there were 400,000 persons in London alone who were paying for treatment as private patients because of their disgust with the panel system. Who inspired such a statement, and with what object ? It will doubtless help in the manufacture of the unpopularity of the Insurance Medical Service, no inconsiderable part of which may be traced to the fact that domestic servants were not excluded from the operation of the National Health Insurance Acts in submission to a manufactured agitation in 1911. I know of no reason for their exclusion which will bear close examination.

### The Attack on the Panel System.

Let us suppose for a moment that there is a successful combined attack of those who desire to get control of the doctors and of those other doctors who have never loved, and never will love, the panel or any other system, supported by those employers—especially of domestic servants—who see in the whole insurance scheme nothing but an objectionable tax. The insurance medical service, as we know it, gets wiped out. What is going to take its place ? Is there to be a reversion to the old catch-as-catch-can methods of getting a doctor ? Are the best interests of the insured person going thereby to be served ? Or are those who are trying to get the doctors entirely under their control going to succeed in their object ? If so, are they then going to secure the services of those doctors who have kept aloof from the panel ? Are they even going to retain the best doctors now in the service, many of whom have so few insured patients on their list that it must be a matter of indifference to them, so far as their income is concerned, whether they keep them or not ? And, again, how are the interests of the insured person going to be served ? It is, after all, for his interest, and not that of the doctors, or the societies, or the Insurance Committees, or the employers, or any other body of persons, that the insurance system exists, however much the interests of those other bodies may be incidentally served.

### Getting Rid of Defects.

Is, then, says the scoffer, this panel system the best of all possible systems in the best of all possible worlds ? I have not said so. I am as little likely as anyone to be under any illusions as to the panel service. It has been my lot, over a term of years, to have had to consider officially the report of every case in the country in which complaints of neglect and failure made against doctors have been investigated, and also to hear unofficially of bad cases in which, because of the difficulty of getting evidence, it has been impossible to proceed ; I have therefore seen the seamy side of the garment. It has, however, been my humble aim to preserve some sense of perspective. The substantial weakness of the present system is that it gives every doctor the right to come on to the panel ; and in the cry of "Free Choice of Doctor" there is much cant. There are too many defects in the working of the system, too many black sheep in the fold, for anyone who has the success of the service at heart to be complacent about it. Those doctors who reply to rather unbalanced criticisms of the service by pointing to the smallness of the number of formal complaints and by extolling the excellence of the service make a mistake. They would do well to follow the lead of Dr. Brackenbury at the Guildhall, who frankly recognised the existence of the defects and said with emphasis that they had got to be put right. In the coming together, with this common aim, of a standing committee, representative of all insurance workers, but small in size, there is much hope for the future of the service. The Committee will consist of six representatives of Approved Societies, six representatives of the doctors, four persons to represent Insurance Committees, and two to represent the pharmacists. It will be at liberty to call into consultation additional representatives of special sections or types of societies particularly affected by any matter under consideration. One matter by no means unworthy of their attention is the securing of a "good Press" in the difficult times that lie ahead. Truth and fair play, it is true, may make dull journalism, but truth and fair play in the end must prevail.

